# Optofluidic Approaches for Enhanced Microsensor Performances

**DOI:** 10.3390/s150100465

**Published:** 2014-12-30

**Authors:** Genni Testa, Gianluca Persichetti, Romeo Bernini

**Affiliations:** Institute for Electromagnetic Monitoring of the Environment (IREA), National Research Council (CNR), Via Diocleziano, 328, Naples 80124, Italy; E-Mails: persichetti.g@irea.cnr.it (G.P.); bernini.r@irea.cnr.it (R.B.)

**Keywords:** optofluidics, sensors, microfluidic, ring resonator, Mach-Zehnder, liquid core waveguides, lab-on-a-chip (LOC), micro total analysis systems (μTAS)

## Abstract

Optofluidics is a relatively young research field able to create a tight synergy between optics and micro/nano-fluidics. The high level of integration between fluidic and optical elements achievable by means of optofluidic approaches makes it possible to realize an innovative class of sensors, which have been demonstrated to have an improved sensitivity, adaptability and compactness. Many developments in this field have been made in the last years thanks to the availability of a new class of low cost materials and new technologies. This review describes the Italian state of art on optofluidic devices for sensing applications and offers a perspective for further future advances. We introduce the optofluidic concept and describe the advantages of merging photonic and fluidic elements, focusing on sensor developments for both environmental and biomedical monitoring.

## 1. Introduction

Optofluidics is a rapidly growing interdisciplinary research field combining micro/nano-fluidics and optics in a very fascinating and valuable way [1]. The concept behind optofluidics is to build up new forms of reconfigurable photonics in which optics and fluidics enable each other. This approach has had a strong impact in the field of optical sensing, leading to the development of a new class of innovative and reconfigurable optofluidic sensors where the fluidic is exploited not only to efficiently handle the sample to be tested, but also as an optical material in order to reconfigure the photonic part of microdevices. Not surprisingly, recent advances in optofluidics have been supported by recent developments in microfluidics, which have led to simpler and precise control of fluids on the micron scale [2], opening new ways to tune microphotonic devices. Additional advantages can be gained by the distinctive properties of fluids interfaces of being naturally smooth and flexible. Some examples of such adaptable devices include fluidic lenses [3] and optofluidic light source [4,5].

In the context of sensing applications, optofluidics arises from the need to hold up very high sensor sensitivity with smaller sample consumption and miniaturized devices. The growing requests of compact devices for complete biochemical and chemical analysis on micron-sized scale have recently seen the development of the so called lab on a chips (LOCs) or micro total analysis systems (μTASs) [6,7]. The optofluidic approaches allow adding further advances to confer optical functions on fluidic elements to such systems. In an optofluidic device microfluidic and optical parts are not separate entities, merely integrated on the same chip, but rather they are strongly tied together, resulting in high interaction efficiency between light and fluids, which is very desirable for sensing applications. The simplest, but not less remarkable example of this mutual correlation is offered by the liquid core optical waveguides, where the fluid is a constituent part of the photonic structure itself [8]. In order to increase device sensitivity, optofluidic sensors are often realized by employing liquid core waveguides or by suitably integrating microfluidic channels along the optical path. In the former case the fluid provides the means to guide the light, enabling a maximized optical coupling across the entire photonic structure. Different approaches for optofluidic sensors consist in exploiting inherent fluidic capability of photonic structure, while preserving evanescent coupling between light and fluids.

Fluids can be efficiently used to transport cells or other biological molecules that are typically suspended in aqueous solutions. In particular, since the detection volumes can be very small (typically of the order of nL), optofluidic microsensors are ideally suited for carrying out single molecule detection [9]. Moreover, the possibility to drive fluids into and out of the fluidic handling system with a continuous piping network makes these devices very attractive for ambient bio-threat detection, where the local environment is continuously tested online [10].

In the following, we describe the recent progress in optofluidic approaches for sensing applications focusing on Italian developments. We firstly offer an overview on optofluidic waveguides, as they often represent the building block of an optofluidic sensor. We present optofluidic devices for spectroscopy and discuss the advantages of the proposed approaches. Optofluidic sensors are also presented and classified on the basis of the basic photonic structure; in particular, interferometric and resonant sensors and their applications are described.

## 2. Optofluidic Waveguides

An optofluidic waveguide can be defined as a structure able to perform optical confinement and transmission of light through a fluid. Optofluidic waveguide represents one of the more significant elements of an optofluidic sensor. As in the approach for solid state optical waveguides, the more obvious solution to confine the light in optofluidic waveguides is based on the total internal reflection (TIR) effect. TIR arises when light propagates, at angles greater than the critical angle, into a material (core) with refractive index n_c_ surrounded by another material with lower refractive index n_cl_ (cladding). The guiding of the light into a liquid core has been proposed in the 1970s, long before the concept of optofluidics was defined, by introducing liquid-waveguide-capillary cells (LWCCs) [11]. In their seminal work, Walrafen and Stone reported intensity enhancement in Raman spectroscopy by a factor of 3000 with respect conventional approaches, using fused quartz capillaries up to 25 m in length, filled with benzene and tetrachloroethylene.

However, as fused silica and other solid materials commonly employed for microelectronic and microfluidic fabrication exhibit higher refractive indexes (1.4–3.5), the condition to fulfill for TIR propagation (n_c_ > n_cl_) poses a severe limitation in the possible liquid core refractive index for optofluidic waveguides. In particular, the use of water solutions (n_H2O_ = 1.33), of great interest in biological applications, is precluded. Most of the optofluidic waveguides described in the following represent the technological and scientific effort to overcome this problem. The first solution was achieved with liquid core waveguides made entirely of Teflon AF2400 (*n* = 1.29 < 1.33). However, despite the fact this material has a suitable refractive index, it also exhibits autofluorescence that contributes to increasing the background noise measurement.

### 2.1. ARROWs Waveguides

**Figure 1.**
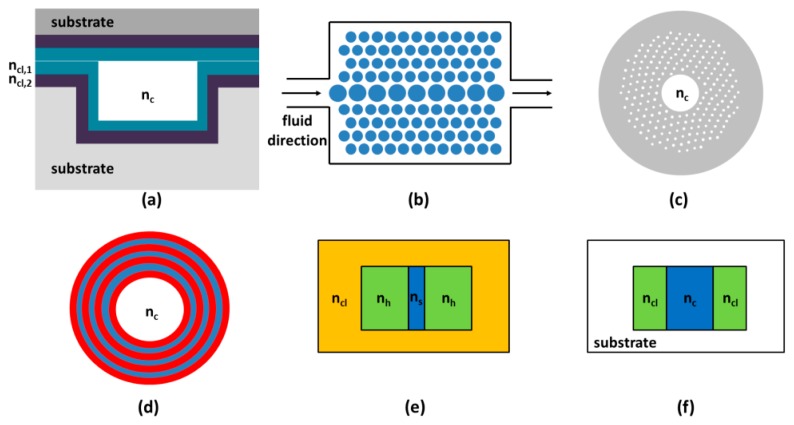
Schematic of some optofluidic waveguides: (**a**) ARROW waveguide; (**b**) Photonics crystal waveguide (top view); (**c**) Hollow-core photonics crystal fiber; (**d**) Hollow-core Bragg fiber; (**e**) Slot waveguide; (**f**) Liquid-liquid waveguide.

Very interesting approaches to confine the light in low index media are based on the use of interference effect in cladding layers able to reflect light back into the core. Liquid core antiresonant reflecting optical waveguides (ARROWs) use such an effect for light propagation [12,13]. A cross section of an ARROW with two layers is represented in Figure 1a. The confinement mechanism is based on the interferences arising in the multilayered structure between reflected and refracted rays in the same way as in Fabry-Pérot mirrors, if specific conditions are fulfilled (antiresonant conditions). The multilayer is designed to operate like a high reflectivity mirror (reflectivity up to 99% can be obtained with four cladding layers) at a specific wavelength. The transmitted spectrum is characterized by a broad spectral range around the design wavelength. ARROWs are fabricated in silicon substrate by employing typical dielectric materials such as SiO_2_ and Si_3_N_4_ for claddings deposition, which are widely compatible with standard silicon process. Recently in the work of Testa *et al.* [14], TiO_2_ deposition by atomic layer deposition (ALD) technique was used to fabricate the high index cladding layer in order to improve the overall optical performance of these waveguides by taking advantage of improved conformality, uniformity and reduced surface roughness which are typical of ALD.

Since 2004, Bernini *et al.* [15] have demonstrated multimode propagation in ARROW proposing a structure able to guide light in low index media (air, gases, and liquids). A very important inherent property of ARROWs is that propagating modes experience attenuation losses depending upon their state of polarization. In particular, the core geometry can be properly chosen in order to select only one polarization state (TE or TM) [12]. Even more significantly, ARROWs can be designed to operate on single polarized optical mode, which allow these waveguides to be applied for designing interferometric optofluidic devices.

Recently, Testa *et al.* proposed a hybrid silicon-poly(dimethysiloxane) (PDMS) liquid core ARROW (h-ARROW) [16]. This configuration differs from traditional full silicon ARROW as it employs a PDMS thin film as top layer. Optical characterizations in agreement with models demonstrated similar optical performances with respect to conventional ARROW and higher degree of adaptability to different optical detection schemes, as the transparence of PDMS allows out-of-plane light detection and excitation. H-ARROWs are very promising for optofluidic devices fabrication as the top polymer part offer greater flexibility for microfluidic integration.

### 2.2. Photonics Crystal Waveguides

Optofluidic waveguides can also be obtained by using photonic crystal (PhC) structures. Also in this case interference is used to guide the light. PhCs are dielectric structures, with sufficiently high refractive index contrast, that are periodically repeated and, in principle, infinitely extended. In analogy with electric field propagation inside a periodic medium, the periodicity of these structures prohibits the propagation of light for frequencies within a band gap (which depends on the periodicity). This periodic arrangement can be obtained in one (1D), two (2D), or three (3D) dimensions. The top view of a 2D structure is shown in Figure 1b.

Since the optical properties of PhCs are highly sensitive to small changes of the thickness and of the refractive index of the composing materials, these structures are considered of great interest for label-free sensors. Hence, by injecting suitable fluids (analyte) into air gaps of a PhC structure, it is possible to identify or to measure optical properties of the fluid under analysis.

The concept of PhC can be used also in hollow-core PhC fibers (HC-PCFs). In HC-PCFs, a hollow core is encircled by a 2D periodic arrangement of holes inside the silica as shown in Figure 1c. A comprehensive work of possible properties and applications of HC-PCFs is in the book of Poli *et al.* [17].

In hollow core fibers based on Bragg guidance mechanism (HC-BFs), the guiding structure is composed by dielectric cladding layers that coat the internal walls of a hollow core as shown in Figure 1d. An interesting structure is obtained by microstructuring the cladding with multiple silica rings joint by nanoscale silica supports. These waveguide, called all-silica hollow-core microstructured Bragg fibers, have been investigated and proposed for biosensing application by Passaro *et al.* [18].

PhC fibers are not limited at hollow core structures as also solid core with microstructured cladding are possible. Among possible solid-core PhC fibers, suspended-core PhC fibers (SC-PhCFs) seem to be very promising for developing efficient biological sensors [19]. The cross-section of a SC-PhCF is composed of a solid rod suspended by three thin supports and it is surrounded by noncircular air-holes. In particular, those waveguides have been investigated for a highly specific DNA biosensor by Coscelli *et al.* [20].

### 2.3. Slot Waveguides

The typical core sizes of a liquid waveguide range from few microns to some hundreds of microns. However, waveguiding properties can be realized on a nanoscale cross section, considering slot waveguides (SWs). This approach has been proposed in 2004 by Almeida *et al.* [21] and subsequently demonstrated experimentally by the same group [22]. The structure consists in a nanometer sized low refractive index (n_s_) slot between two region with high refractive index (n_h_). The whole structure is surrounded by a cladding with low refractive index n_cl_. A typical cross section of a SW waveguide is shown in Figure 1e.

In a SW, an enhanced and confined optical field is observed in the low refractive index (n_s_) material slot even if light propagates exploiting TIR. As the normal component of the electric displacement field D has to be continuous at the interface of two dielectric materials, the normal component of the electric field E experiences a discontinuity at this interface (D = n^2^ε_0_E where n and ε_0_ are the refractive index and the vacuum permittivity, respectively). In particular, the normal component of E has to be higher at the low-index side and lower at the high-index side. In structures with high index contrast as SW, this discontinuity is particularly significant and up to 30% of the total modal power is confined in the slot region in a 100 nm narrow core considering a high index contrast system such as Si/air [21].

The small size where the electric field is confined (which is comparable to the decay length of E) and the high intensities available in a SW, make those structures very attractive for optofluidic applications. For this purpose, extensive investigation has been carried out in order to optimize the sensing properties of SW. In particular, in the works of Passaro *et al.*, the optimization of silicon-on-insulator SW has been carried out considering also the influence of fabrication tolerances in order to enhance optical sensing [23–25].

Silicon nanocrystals-based sandwiched SWs has been theoretically investigated by Passaro *et al.* [26]. By changing the upper layer thickness only of few nanometers, the authors demonstrated, for the first time, the possibility to create or to annihilate an optical solitons inside those waveguides.

Moreover, Bettotti *et al.* [27] explored the possibility of using SWs realized by considering very low refractive index materials such as for instance, polymers. Since polymers are particularly flexible materials (and transparent at visible wavelengths) also allowing surface functionalization, the proposed SWs are very promising optical waveguides in sensors designing.

Recently, a novel configuration called Layer-slot (L-slot) has been proposed by Testa *et al.* [28], which is able to operate in the visible wavelength range. In this range the optical absorption of water is about three orders of magnitude lower than in IR region, reducing optical losses and thermal heating. In addition, the proposed configuration contributes to simplify the SW fabrication process as it is based on a deposition step instead of the conventional etching processes, hence high-resolution techniques are not required.

### 2.4. Liquid-Liquid Waveguides

Another approach to realize an optical waveguide consists in using liquids cladding inside a larger fluidic channel. In this approach, by adopting a suitable flow rate, the two liquids will flow in laminar condition and the only diffusion process will be responsible of the mixing of the fluids. This means that the waveguiding properties are ensured by TIR, until the cladding refractive index n_cl_ remains smaller than the core refractive index n_c_ and the cladding thickness is reduced to a few tens of microns. A cross section of such a liquid-liquid waveguide (L2) is shown in Figure 1f. The L2 approach has been successfully demonstrated by Wolfe *et al.* [29] employing CaCl_2_ water solution (*n* = 1.445) as core and water as cladding, both embedded in PDMS (*n* = 1.4).

Manipulating the flow rate and the liquids composition, it is possible to tune many optical properties, such as for instance refractive index contrast, size and loss/gain properties. The attainable optical tunability is one of the more interesting properties offered by L2 waveguides. The L2 concept has been extensively proposed in a 2D geometry, *i.e.*, the liquid core is bordering on the liquid claddings only in one transverse direction, whereas in the other transverse direction, the liquids are surrounded by solid materials. This circumstance leads to detrimental effects on the waveguiding properties, preventing the possibility to use water and very dilute water solution as core materials. A possible solution has been proposed by Bernini *et al.* [30] by adopting a hybrid approach in which L2 waveguide is accomplished in the microfluidic channel of an ARROW waveguide, providing light confinement in both of the transverse directions. This same difficulty has been differently solved by exploiting 3D hydrodynamic focusing effect [31]. In this 3D approach, the liquid core is completely surrounded by the liquid cladding using fluid flows from concentric glass capillaries. Since the first implementation of a 3D hydrodynamic focusing effect, proposed by Takiguchi *et al.*, several improvements have been proposed [32–34]. In 2012, Testa *et al.* [35] numerically and experimentally demonstrated an innovative scheme, with reduced input channels, for 3D hydrodynamic focusing which is able to produce a tunable and circular liquid core placed in the center of the focusing channel.

### 2.5. Jet Waveguides

The last optofluidic waveguide we describe is based on a simple and clever idea coming from the past. The first demonstration of an optofluidic waveguide was given almost two centuries ago, when the waveguiding nature of a water jet was discovered and explained by Colladon [36] as due to TIR at the water-air interface. Microfluidic liquid jets are regular cylinder of fluids in air that can be obtained by injecting the liquid into a channel or a capillary at a specific range of flow rate, depending on the channel size diameter. This regular shape in the jet is observed up to a specific length (breakup length).Beyond this length, the jet breaks up into drops.

As other liquid core waveguides, jet waveguides exploit TIR for light propagation. However, a very high refractive index contrast between the core (liquid) and cladding (air) leads to a very high numerical aperture (NA = 0.88 for water core). Hence, in principle, liquid jets exhibit significant collection efficiency. The advantages of these simple but effective optical waveguides have been neglected until recent times [37] when the group led by Bernini proposed an experimental configuration able to overcome the typical complications arising in conventional approaches. Liquid core waveguide with solid or liquid cladding are usually surrounded by solid structures that are potential sources of scattering (by surface roughness) or autofluorescence. Beside the advantage related to its collection efficiency, in a jet waveguide, the absence of solid walls to contain the liquid waveguide and the smoothness of the liquid/air surface enable very low background signal arising from scattering.

## 3. Optofluidic Devices for Spectroscopy

Spectroscopy methods like absorption, fluorescence, and Raman are mostly used as optical detection methods, as they enable highly selective and sensitive detection of analytes at a very low concentration level. However, device miniaturization has the unavoidable effect of reducing the interaction length of light with fluid due to the overall device size reduction, thus limiting the sensor sensitivity [38]. The use of optofluidic waveguides or microfluidic channels designed to catch directly the optical probe enables an enhanced optical interaction with sample under analysis and hence an increased sensitivity. Optofluidic chips, besides retaining the advantages of high optical sensitivity, have the potential to address the growing needs of reduced device size, low sample consumption and field deployability. In the following we present the recent progress in development of optofluidic devices for spectroscopic analysis of fluids. Among others spectroscopic methods, fluorescence and Raman spectroscopy have been demonstrated as very sensitive techniques; analysis and detection of molecules on the single particle level has also been demonstrated by implementing these techniques on optofluidic chip [39,40].

**Figure 2.**
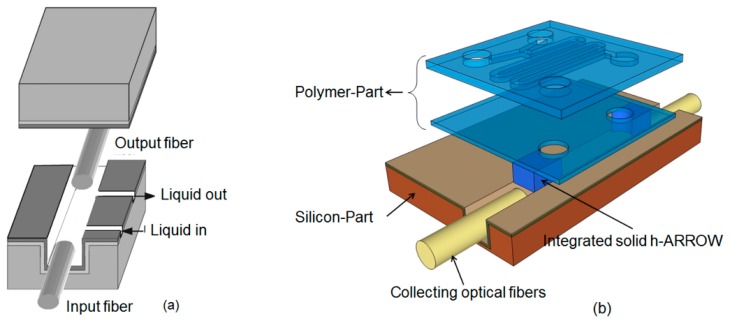
(**a**) Schematic of the microfluidic sensor for bulk refractometer RI measurements. Reprinted with permission from [42] (Copyright (2004) OSA) (**b**) Schematic drawing of the hybrid optofluidic platform. Reprinted with permission from [45] (Copyright (2014) OSA.)

In this scenario liquid core ARROWs play a very remarkable role. Liquid core waveguides have the inherent advantage of exposing the liquid analyte to the entire optical power as both light and liquid are being guided through the same channels. As a consequence, maximized optical sensitivity in bulk sensing distinguishes these waveguides among others. Moreover, like optofluidic slot- and PC-waveguides, ARROWs are photonic structures that could be realized using silicon technology and hence they are highly attractive for planar optofluidic integration [13]. The potentialities of these waveguides in the field of optical sensing have been explored since the first demonstration of their guiding capability [39,41]. In the work of Campopiano *et al.* [42], an integrated optical bulk refractometer based on multimodal liquid ARROW has been demonstrated. The waveguide itself constituted the optical sensor, with input and output optical fibers directly inserted in the waveguide hollow core for the connection with off-chip optical source and detection system. The operating principle was based on the shift of the minimum of the transmitted spectrum upon variation of the refractive index (RI) of the fluid filling the core. A linear response and a sensitivity of about 555 nm/RIU were measured, with an LOD of 9 × 10^−4^ refractive index unit (RIU). Fluid injection/ejection in the microfluidic core was achieved via lateral channels, orthogonally crossing the core (Figure 2a). The potential for use in spectroscopy of the ARROW microfluidic channel has been demonstrated by using the same configuration to realize a long path absorbance cell, for colorimetric determination of concentration of specific protein in water solution [43]. In particular, by using Bradford assay, authors were able to detect bovine serum albumin with an LOD of about 1.5 × 10^−3^ mg/mL. Certainly these sensors, together with other existing examples in literature [39,41], can be considered as starting points for ARROW based optofluidic chip development for spectroscopic measurements. However, further advances can be gained by optofluidic approaches, obtained through the integration of microfluidic components that allow a more precise control and handling of fluid sample [44].

Recently, in the work of Testa *et al.*, an optofluidic platform based on hybrid liquid core ARROW waveguides for fluorescence spectroscopy of liquids has been presented [45]. The chip was assembled in a modular structure, with the upper polymeric part including solely microfluidic functionalities (Figure 2b). The optical part has been realized developing a polymer-silicon hybrid solution in order to form a fully integrated platform connected with the upwards microfluidic system. Solid core hybrid ARROWs (solid h-ARROWs) have been suitably integrated in the polymeric part in a self-aligned configuration with the liquid core waveguides containing the sample to be tested. This hybrid solution allows us to seal the liquid h-ARROW and to couple light from/toward the exciting/collecting off-chip optical fibers. The presented approach is unique among other as hybrid integration was implemented for the fabrication of the both photonic and fluidic elements on the same chip. A first prototype has been fabricated with integrated passive micromixer as microfluidic element. The sensing performance of the device was tested by performing fluorescence measurements at different concentration, controlled by means of the integrated micromixer; an LOD of 2.5 nM was demonstrated. The proposed modular approach represents the major step toward a truly hybrid optofluidic chip and offers prospects of high functional flexibility inherent to optofluidics, as the microfluidic part can be easily replaced and adapted to different detection schemes.

Planar optofluidic chip has also been developed in bulk fused silica by using femtosecond laser technology (FLT). FLT is a well-established fabrication technique that exploits refractive index modifications induced by focused femtosecond pulses to inscribe photonic circuits in glass substrates; it has also been demonstrated as a powerful tool for microfluidic chip fabrication [46]. In the work of Osellame *et al.*, it has been demonstrated that FLT can be exploited to fabricate both microfluidic channels and optical waveguides on the same fused silica substrate [47], paving the way towards the realization of monolithic optofluidic devices. Even if polymer materials are going to became more and more preferred for LOC fabrication, glass is still used in some applications. Based on FLT, an optofluidic chip for dual-wavelength fluorescent DNA analysis is presented in the work of Dongre *et al.* [48]. A microfluidic channel network has been suitably integrated with crossing exciting/collecting optical waveguides by means of FLT on a fused silica glass slide, towards an on chip fluorescence excitation of DNA molecules. Capillary electrophoresis has been implemented on the chip for separation of fluorescently labeled-DNA molecules. Dual point, dual-wavelength laser induced fluorescence has been used to optically resolve separation of two equally sized DNA molecules, which could not be electrophoretically distinguished. Similar device design has also been used to demonstrate a proof of principle color-end-labeled DNA identification by means of modulation-frequency-encoded multi-wavelength excitation [49]. The potential of the proposed chips makes them very interesting, in particular for prospective use in point-of-care diagnostics.

Spectroscopic optofluidic devices are also finding significant applications in the field of flow cytometry for cell counting, analysis and sorting. In the last years, many research efforts have been dedicated to find new solutions to the question of handling particles at a single level on planar miniaturized structures [50]. Like in a bulk cytometer, cells must be organized in a single line to pass through the detection region, where a collimated optical beam can provide single cell interrogation at a time. Microfluidic strategies for arranging particles in a single line are mainly based on hydrodynamic focusing effect. In this case, a sample flow with suspended cells is wrapped and squeezed by suitable sheathing fluids. Miniaturization of flow cytometers with planar approach is a challenging objective that has seen a growing interest by the research community in the last years [34,51]. An integrated micro flow cytometer has been proposed in the work of Bernini *et al.* [52]. A hydrodynamically focused stream of fluorescently-labeled human T leukemia cells (Jurkat) has been accomplished in the optofluidic channel of an ARROW waveguide. The authors exploit the dual ability of an ARROW waveguide to both serve as a microfluidic channel for flow focusing and efficiently confine the excitation light for cell interrogation. Optical fibers for fluorescent detection have been integrated on the same platform, orthogonally crossing the focusing channel to reduce the pump contribution to detected signals. The same samples were also tested by using a bench-top flow cytometer. Qualitative accordance has been achieved between results, demonstrating the validity of the proposed approach.

Sorting of cells is another very useful functionality in many biological applications. It can be applied to isolate cells with specific properties like particular size and weight. By using FLT, an optofluidic device implementing an integrated fluorescence-activated cell sorter has been demonstrated [53]. The device comprises a microfluidic channel for cells flowing and two integrated optical waveguides for cells fluorescent excitation and sorting via optical force, respectively. Optical waveguides were arranged in an orthogonal scheme with the flow channel. The optical force is activated when fluorescent cells pass through the interrogation region produced by the excitation waveguide, where a CCD camera for fluorescent cells recognition is located. Isolation of subpopulations with high selectivity from heterogeneous samples has been demonstrated by authors. A similar device employing two facing optical waveguides, crossing the microfluidic channels, has been demonstrated for optical trapping of single red blood cells by means of counterpropagating dual beams traps [54]. These results are very promising in the view of realizing micro-sized devices for manipulation of cells at the single particle level.

**Figure 3.**
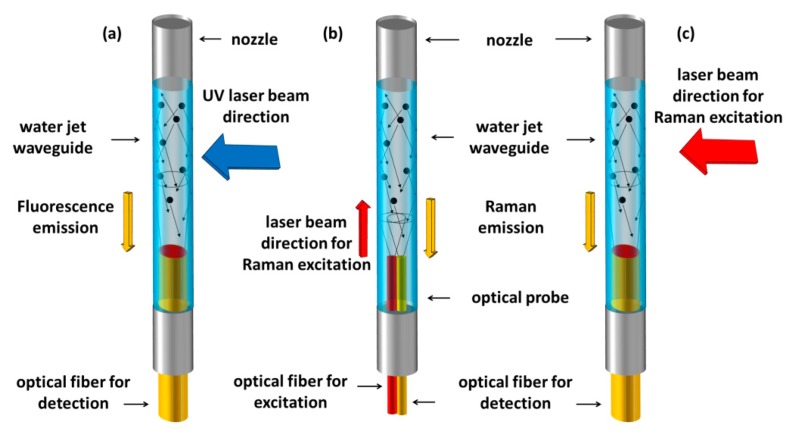
Schematic of the optofluidic device based on a water jet waveguide. (**a**) Configuration for fluorescence spectroscopy; (**b**) Configuration for Raman spectroscopy with two side by side optical fibers and (**c**) with orthogonal excitation.

Despite the fact water jet waveguides were the first demonstration of an optical waveguide, only in recent years, have spectroscopic sensors based on this approach been proposed by Persichetti *et al.*, for both Raman spectroscopy (RS) [55] and fluorescence spectroscopy (FS) [37]. In these optofluidic sensors, the liquid jet acts as the liquid under analysis and as an optical waveguide. Fluorescence or Raman signal, experiencing TIR, is efficiently collected by the liquid jet waveguide (which owns a very high numerical aperture as shown in Subsection 2.5) and it is delivered towards the detector by means of a multimodal optical fiber which is directly coupled with the jet by means of a self-aligned configuration (Figure 3a). As organic compounds exhibit autofluorescence when excited by UV light [56], the authors detected several organic compounds (water pollutants) and bacteria cells at very low limit of detection. For instance, polycyclic aromatic hydrocarbons like naphthalene have been detected in water solutions at 2.2 ppb level, and monoaromatic hydrocarbons at levels in the 0.1–2 ppm range. FS based on autofluorescence offers several advantages in sensoristics for continuous online monitoring. For example, there is no longer any need for reagents or sample pretreatment. The performances attainable with this approach are often below levels allowed in drinking water according to US Environmental Protection Agency (EPA), showing that the sensor is suitable for environmental monitoring activity, in particular, for early warning systems [37].

The same waveguiding nature of a liquid jet can also be conveniently exploited for designing an optofluidic Raman sensor. In a recent work of Persichetti *et al.* [55], two different experimental approaches for RS have been proposed, each of them possessing a specific advantage. In order to exploit the maximum available volume, *i.e.*, the whole liquid jet, a probe consisting of two side by side optical fibers has been arranged (Figure 3b). One fiber was used to deliver laser excitation in the liquid waveguide and the other was used to collect the Raman signal. In order to provide the optimal matching between the jet size diameter and the optical fiber used to collect the Raman signal, an experimental configuration similar to the one successfully employed in FS has been adopted (Figure 3c). By exciting the liquid waveguide in the orthogonal direction with respect to the flow direction, it is possible to use a collecting optical fiber with a diameter size close to the jet diameter, ensuring minimal diameter mismatching losses. Both of the configurations have shown high performance detection in measurements performed using water-ethanol solutions, leading to competitive results with respect more complex optofluidic approaches. For instance, in the measurement configuration with optical fiber excitation, the authors observed an LOD = 0.022% of ethanol concentration which is a competitive value with respect an LOD of 0.2% reported using FT-Raman spectroscopy [57] or the 1% level reported for hollow core based sensor [58].

## 4. Optofluidic Interferometer

Integrated interferometric devices are widely applied in sensing applications and have been demonstrated mainly in two optical configurations: Mach-Zenhder (MZ) and Young interferometers [59]. Integrated interferometers have proved very high sensitivity for biochemical and biomedical detection, which is the reason why innovative optofluidic architectures have been explored, to provide these devices with the further improvement arising from microfluidic integration.

Unlike Michelson interferometer which requires optical mirrors to build the optical path for beams recombination, MZ design lend itself to chip integration more easily, as it is demonstrated by the large amount of publications appeared in the last decades. However, using the flexibility given by optofluidics, a Michelson interferometer has been demonstrated using a droplets grating created by using two immiscible liquids [60]. Optofluidic MZs have mainly been realized either by employing liquid core waveguides or by inserting microfluidic channels along the optical path of a solid core interferometer [61]. Nevertheless, novel solutions arise from optofluidic that have led to innovative schemes which do not necessarily require splitting a beam into two separated arms to achieve phase difference. Optofluidic MZ schemes for sensing applications have been demonstrated where a phase delay is induced on a portion of a beam propagating across a liquid/air interface [62] or a liquid/solid interface [63], with a single collimated light beam.

**Figure 4.**
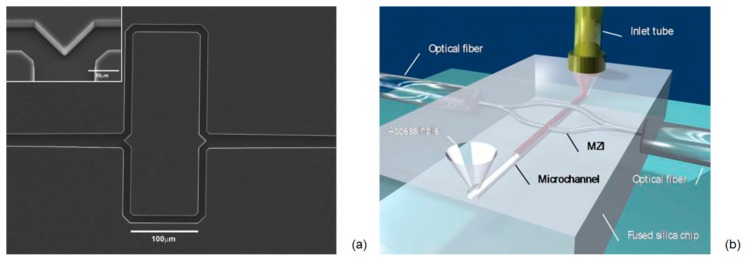
(**a**) SEM image of the second generation LC-MZI. Reprinted with permission from [65] (Copyright (2010) OSA) (**b**) Schematic of the femtosecond-laser-fabricated microfluidic channel and integrated MZI. Reprinted with permission from [66] (Copyright (2010) RSC Publishing).

A liquid core MZ (LC-MZ) has been proposed by Bernini *et al.*, based on liquid core ARROW waveguides (LCAs) [64]. Basically, the design consists of an input LCA waveguide that is split into two separate interferometer arms before recombining to form the output waveguide. Since the core refractive index is set by the fluid flowing in the entire device, phase difference between the interfering beams is achieved by employing an asymmetric configuration, with two arms having different physical length. In a first generation LC-MZI a Y-junction and cosine-shaped arms have been employed in order to reduce the bending losses. However, the asymmetric geometry explored in the first prototype causes a degradation of the device's performances due to the poor visibility of the interferometer. The visibility of a MZI is strictly related to the polarization and the intensity balancing between the beams emerging from the two arms of the interferometer. In the first LC-MZI prototype, the power unbalancing comes from the difference in curvature between the two arms, which causes different bending losses. A great improvement of interferometer's visibility has been demonstrated by Testa *et al.*, with the second generation LC-MZI [65]. In order to achieve a better visibility factor, the shape of the arms has been adjusted in order to balance, as well as possible, the intensities of the interfering beams. The device is constituted by an input straight LCA ending in a tapered T junction that splits equally the input power into the two arms of the interferometer (Figure 4a). With the proposed design the path-length difference between the two arms is attained by modifying the length of the straight section, instead of altering the curvature between the two arms. As a consequence, the power unbalancing is minimized and a best visibility of 0.99 was demonstrated. About sensing capability, a best value of bulk sensitivity of Δn = 1.6 × 10^−5^ RIU has been estimated. The device exploited a large refractive index tunability, distinctive of optofluidic microsystem; in fact, the guiding condition in the LCA is satisfied for a quite large index range n_c_ = 1.32/1.45, that corresponds to the detection range of the sensing device. To the best of our knowledge, the proposed LC-MZIs were the first examples of integrated MZ interferometers composed entirely of liquid core waveguides. Compactness and fluidic-photonic cross correlation are remarkable in the proposed LC-MZI; however, further improvements can be envisioned, achievable by means of hybrid approaches which benefit from simpler and faster fabrication procedures for incorporating advanced microfluidic functionalities into the chip.

Other interesting optofluidic approaches have been developed integrating solid-core waveguides with liquid-core waveguides or microchannels [61]. Femtosecond laser technology (FLT) has recently offered the opportunity for 3D architectures of microchannels and optical waveguides, without the requirement for top sealing. In an earlier work [66], Crespi *et al.*, exploited FLT to fabricate a MZI with the sensing arm orthogonally crossing a microfluidic channel for sample flowing and the reference arm passes over it (Figure 4b). The authors claimed an increased sensitivity due to direct optical coupling between the propagating waveguide mode and the fluid sample in the crossing region. In this configuration the interaction length is limited by the width of the microfluidic channel and calibration of the MZI device, carried out by using glucose solutions at different concentrations, revealed a bulk sensitivity of about 10^−4^ RIU. Despite the moderate sensitivity, the proposed approach retain the merit of high level of optofluidic integration and of very promising future prospects for direct on-chip integration of photonic and fluidic devices. After demonstrating the proof of concept of label-free sensing in an optofluidic MZI entirely fabricated by FLT, a further step toward application of their approach to LOC devices is performed by integrating the MZI in a commercial LOC for capillary electrophoresis. In this case the authors demonstrated an LOD for monopeptide of 10 mM, which makes the device very promising for assaying the products of chemical reactions.

## 5. Optofluidic Resonators

Optical microresonators are very interesting and promising photonic structures for high sensitivity miniaturized biological or biochemical sensing [67]. The main advance of these structures is the recursive interaction between light and sample, achieved thanks to the resonant recirculation of light in the cavity. This phenomenon has the effect to enhance the coupling strength of light with the sample and, in the sensing applications, to improve sensitivity. Optical resonator architecture allows overcoming the typical limitation occurring in a MZI, *i.e.*, a reduced interaction length limited by the physical length of the sensing path. The effective light-sample interaction length L_eff_ is governed by L_eff_ = Qλ/2 πn where Q is the ring resonator quality factor, λ is the resonant wavelength, and n is the optical mode effective refractive index. Optofluidic resonators for sensing applications have been recently demonstrated by using the same fluids as the only element to realize the resonant cavity, such as microdroplet resonators [68], or by efficiently including fluidic capability in the photonic structure.

**Figure 5.**
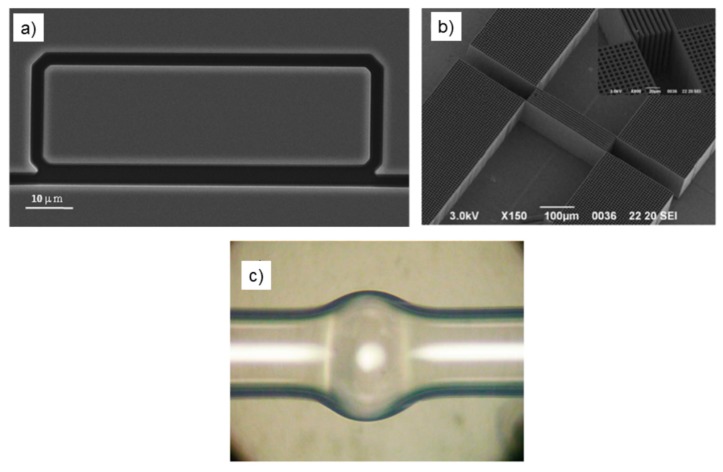
SEM image of (**a**) the integrated optofluidic ring resonator based on liquid ARROWs. Reprinted with permission from [69] (Copyright (2010) AIP); (**b**) PhC-OFM with PhC structure height and width of 90 μm and 130 μm, respectively. In the image are also shown the fiber grooves. Reprinted with permission from [74] (Copyright (2012) RSC Publishing); (**c**) Optical image of microbubble with outer diameter of ∼340 μm. (Reprinted with permission from [77] Copyright (2011) OSA.)

A very interesting approach to realize optofluidic resonators is based on the use of liquid core waveguides. A high level of integration can be achieved with this approach as the optical and microfluidic functionalities are closely combined, as the waveguide core serves also as microfluidic channel. An optofluidic ring resonator (ORR) for sensing application based on liquid core ARROW has been demonstrated by Testa *et al.* [69]. The ARROW's liquid core itself is used to give shape to the resonant cavity loop with great advantage in terms of compactness and integration. 90°-bent ARROW waveguides have been used to connect straight optofluidic channels in order to form a rectangular shaped ring resonator (Figure 5a). The coupling of light from input waveguide to the resonant cavity is achieved by means of a multi-mode interference (MMI) liquid core ARROW with a 50:50 splitting ratio. Moderate Q factor of the order of 10^3^ were experimentally demonstrated, opening the way for further optimization and better performances. ARROWs are leaky waveguides and require an accurate design in order to minimize the propagation losses, especially in the curved sections, where higher order modes are excited. Recently, the possibility to use a hybrid version of ARROWs offered the opportunity to improve the microfluidic functionalities of the ORR by integrating microfluidic elements in the polymer part, for manipulating and delivering fluids into the resonant cavity. A hybrid version of the ARROW-based optofluidic ring resonator could take advantage of low cost and fast fabrication technique nowadays available to handle polymer materials. In order to compensate for slightly higher propagation losses arising in the hybrid version, different ARROW configurations have been explored by varying the number of cladding bilayer with the aim of improving the confinement effect in the liquid core. A detailed design procedure, based on the optimization of each optical element composing the hybrid ring has been reported [70]. Overall the optimization criterion was the requirement of low propagation losses for a high sensitivity and high quality factor ring resonator. Quality factor up to 4 × 10^4^ has been estimated by simulations and bulk RI detection limit of Δn_min_ = 3.7 × 10^−6^.

Optofluidic resonators have been also fabricated using photonic crystals. These structures are a powerful building block for optofluidics as the cavity holes, designed to provide strong light confinement, can also be exploited for fluid flowing [71]. The group of Barillaro *et al.*, have recently demonstrated that a high-aspect ratio vertical silicon/air walls 1D PhCs is a very powerful tool for optofluidic applications [72]. Basically, by exploiting the air gaps as microfluidic cavity, they developed a first generation prototype of an integrated refractive index optofluidic sensor by using a 1D PhC sealed with a borosilicate glass cover with integrated a fluid inlet for liquid injection into the air-gaps. A bulk sensitivity of 1049 nm/RIU at λ = 1.55 μm has been demonstrated, which is well compared to sensitivities of state-of-the-art integrated refractive index sensors. As it has been envisioned by the group, high aspect ratio 1D PhCs are very promising key structures for the realization of more sophisticated optofluidic microsystem [73]. A second generation PhCs optofluidic microsystem (PhC-OFM) has been realized by the group, with integrated fluidic and optical sections on the same chip [74] (Figure 5c). By exploiting electrochemical machining (ECM) technology, vertical silicon walls with height comparable with standard optical fiber diameter were fabricated. Moreover grooves etched in silicon were suitable integrated, thus allowing a simple alignment procedure between the PhC structure and the readout optical fibres. Fluidic section for handling injection of fluids in the 1D PhC air gaps comprise reservoirs/microfluidic channels integrated in silicon. A best bulk sensitivity value of 670 nm/RIU and a best limit of detection of the order of 10^−3^ RIU were demonstrated. A very promising proof of concept about the biosensing potential of PhC-OFM has been also demonstrated by carrying out an immunoassay for C-reactive protein, paving the way to the opportunity of realizing a fully integrated PhC optofluidic biosensors.

The capillary-based optofluidic resonator (CORR) represents another interesting architecture where the fluidic and photonic elements are elegantly merged [75]. The thin wall of a silica microcapillary forms a ring resonator able to support whispering gallery modes (WGMs) and, at the same time, has the function of a microfluidic core for fluid flowing. The WGM is a surface mode which evanescently couples with liquid sample flowing through the capillary or with the analyte on the interior surface wall. Capillaries that have very smooth sidewalls can be used, thus allowing high Q factors of the order of 10^6^. The sensing capability to bulk RI changes in the core has been successfully demonstrated and a bulk detection limit of approximately 10^−7^ RIU has been proved. Detection of biomolecules adhered on the interior wall surface has been also carried out with a detection-limit of approximately 1.6 pg/mm^2^ [76]. CORRs are very promising devices, however they suffer from some limitations that have slowed their integration onto lab on chip platform. While the microfluidic integration is straightforward, being inherent to capillaries, main photonic elements, such as the waveguide to excite WGMs, need to be fabricated separately and then included on the same platform. This step is basically hindered by low tolerance to angular misalignments of the optical excitation system, due to the poor 2D light confinement of capillary WGMs. In this contest, recent advances have been made to simplify the WGMs optical excitation. In the work of Berneschi *et al.* [77], a novel fabrication technique based on arc discharge has been successfully used to produce highly controllable microbubbles in a glass capillary with Q factors larger than 10^7^ (Figure 5c). In this way, besides the intrinsic microfluidic capability of a glass capillary, relaxed tolerance in optical excitation can be achieved due to 3D confinement of WGMs supported by the microbubble. Bulk RI sensing performance of 0.5 nm/RIU and a detection limit of 10^−6^ have been experimentally measured by the group.

## 6. Conclusions

Optofluidics is still a relatively young research field that has seen rapid progress in the last few years as demonstrated by the large number of papers dedicated to this concept. As illustrated by the discussed examples, optofluidic approaches offer bright prospects for sensor development. Italian researchers have played an important role in this context, establishing very interesting strategies to design optofluidic devices for enhanced sensing performances. Major efforts in this direction have been made by developing innovative design and fabrication solutions able to improve, and potentially maximize, the coupling strength between light and fluids. These strategies include the use of liquid core waveguides or fluid filled photonic bandgap structures to realize the photonic architecture, and the monolithic integration of microfluidic channels crossing optical waveguides in glass substrate. Many valuable applications have been demonstrated using these devices, ranging from environmental sensing to biosensing, like detection of water pollutants for drinking water monitoring or DNA investigation as relevant in genetic diagnostics. The obtained results are very competitive in the international scenario and further developments could be envisioned in the future. However, most of the research done so far has addressed only some of the requirements for a complete LOC optofluidic device, *i.e.*, the fluidic-photonic mutual integration for enhanced sensitivity and functionality. Optical components for interrogation and signal collection continue to consist mainly of off-chip bulk components. Great efforts need to be devoted in the near future to solve this aspect, otherwise it will continue to hinder the use of these devices for point-of-care-diagnosis or *in situ* analysis in environmental monitoring. One possible solution to this concern, where Italian researchers are also involved, is to use hybrid approaches in the manufacturing process in which silicon is combined with polymer or glass materials. Nowadays, the use of silicon substrates makes the integration of photonic source and detection system straightforward. Also glass-based substrates manufactured by FTL have demonstrated a great potential for optofluidic integration. The possibility to use hybrid approaches to fabricate the microfluidic system and/or parts of the photonic structure could offer great advantages in terms of costs of the involved materials and technologies, without compromising the optical performance of the resulting devices. This approach could significantly increase the portability of such microsystem on the way to the realization of reliable LOCs, enabling their large-scale diffusion and commercialization.
